# Shape and Structure Formation of Mixed Nonionic–Anionic Surfactant Micelles

**DOI:** 10.3390/molecules26144136

**Published:** 2021-07-07

**Authors:** Michael Ludwig, Ramsia Geisler, Sylvain Prévost, Regine von Klitzing

**Affiliations:** 1 Soft Matter at Interfaces, Institute for Condensed Matter Physics, Technical University of Darmstadt, D-64289 Darmstadt, Germany; ludwig@fkp.tu-darmstadt.de (M.L.); geisler@fkp.tu-darmstadt.de (R.G.); 2 Large Scale Structures Group, DS/LSS, Institut Laue-Langevin, CEDEX 9, 38042 Grenoble, France; prevost@ill.eu

**Keywords:** small-angle neutron scattering, surfactant, micelles, mixed micelles, Tween20, polysorbate 20, BrijL23, Brij 35, SDS

## Abstract

Aqueous solutions of a nonionic surfactant (either Tween20 or BrijL23) and an anionic surfactant (sodium dodecyl sulfate, SDS) are investigated, using small-angle neutron scattering (SANS). SANS spectra are analysed by using a core-shell model to describe the form factor of self-assembled surfactant micelles; the intermicellar interactions are modelled by using a hard-sphere Percus–Yevick (HS-PY) or a rescaled mean spherical approximation (RMSA) structure factor. Choosing these specific nonionic surfactants allows for comparison of the effect of branched (Tween20) and linear (BrijL23) surfactant headgroups, both constituted of poly-ethylene oxide (PEO) groups. The nonionic–anionic surfactant mixtures are studied at various concentrations up to highly concentrated samples (ϕ ≲ 0.45) and various mixing ratios, from pure nonionic to pure anionic surfactant solutions. The scattering data reveal the formation of mixed micelles already at concentrations below the critical micelle concentration of SDS. At higher volume fractions, excluded volume effects dominate the intermicellar structuring, even for charged micelles. In consequence, at high volume fractions, the intermicellar structuring is the same for charged and uncharged micelles. At all mixing ratios, almost spherical mixed micelles form. This offers the opportunity to create a system of colloidal particles with a variable surface charge. This excludes only roughly equimolar mixing ratios (*X*≈ 0.4–0.6) at which the micelles significantly increase in size and ellipticity due to specific sulfate–EO interactions.

## 1. Introduction

Surfactants are widely used in various industrial, technological or domestic applications [1]. Mixtures of surfactants typically form self-assembled mixed micelles, with their properties differing from those of the individual surfactants [2,3]. This useful feature allows for tailoring the physicochemical properties, such as reduction in the critical micelle concentration, increased foaming, beneficial rheological properties and many more [4,5,6]. Surfactant mixtures, including nonionic surfactants, are often used as emulsifiers in food, cosmetics and pharmaceuticals since they are usually biocompatible and have low critical micelle concentrations [7].

Understanding the structure and properties of surfactant mixtures is important not only for application, but also in fundamental science: alkyl ethers with headgroups constituted of poly-ethylene oxide (PEO) groups (often abbreviated as $C_{n}E_{m}$) are often used to study properties of mixed surfactant solutions [8,9]. Their chemical structure is a great model system, allowing to vary the hydrophilic/hydrophobic character of the nonionic surfactant systematically when mixed with other surfactants.

Numerous studies report on mixed surfactant systems from mixing nonionic $C_{n}E_{m}$ surfactants and the well-known anionic surfactant sodium dodecyl sulfate (SDS): experiments include various techniques, such as tensiometry [10,11,12], conductometry [13,14], fluorescence microscopy [15], light scattering [16,17,18], nuclear magnetic resonance [19,20], electron spin echo modulation [21], and capillary electrophoresis [22]. Theoretical models for the thermodynamic properties of mixed micelles have been proposed based on experimental observations of the micellation characteristics [8,23,24].

Small-angle neutron scattering (SANS) provides a more detailed structural characterisation of self-assembled mixed micellar structures. Many works report on SANS experiments on mixed SDS/$C_{12}E_{m}$ surfactant solutions, with the number of EO groups varying between m = 3–8 [25,26,27,28,29,30,31]. Besides information on micelle properties, SANS experiments yield information on the role of headgroup electrostatics and on the steric effects on the free energy of micelle formation.

The literature still lacks a structural investigation on SDS/$C_{12}E_{m}$ mixed micelles with large, hydrophilic, nonionic surfactant headgroups (m > 20). Additionally, little is known on the difference between nonionic headgroups of one linear PEO chain and multiple, branched PEO chains per headgroup when mixed with SDS. Furthermore, the behaviour at high surfactant concentration, at which strong and maybe attractive interactions between the mixed micelles may occur, is yet to be answered.

In order to close this gap, we investigate the aggregation behaviour of mixtures of nonionic surfactants and well-known anionic sodium dodecyl sulfate (SDS) surfactants. As nonionic surfactants, we use the commercially available nonionic surfactants BrijL23 and Tween20 since the combination of both allows to investigate the effect of branched (Tween20) and linear (BrijL23) hydrophilic headgroups with a similar number of EO groups. Mixed surfactant solutions are studied at various concentrations and various mixing ratios, using SANS. These data reveal the morphology of single micelles as well as their effective radii and aggregation number. Moreover, analysis of the structure factor provides the volume fraction and surface charge of the micelles.

## 2. Materials and Methods

### 2.1. Materials

**Figure 1:**
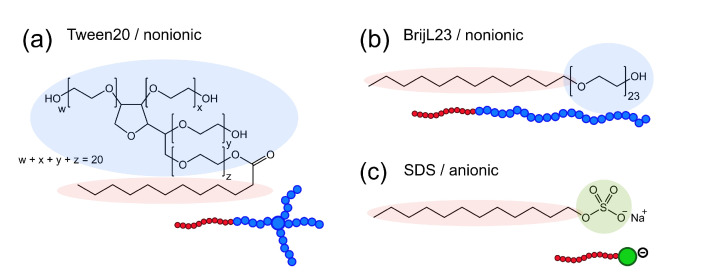
The molecular structures and schematic drawings of the surfactants used in this study: (**a**) nonionic Tween20, (**b**) nonionic BrijL23, and (**c**) anionic SDS. The hydrophobic alkyl chains are highlighted in red. The hydrophilic headgroups are highlighted in blue and green for nonionic polyethyleneoxide (PEO) groups and the anionic sulfate group, respectively.

**Figure A1:**
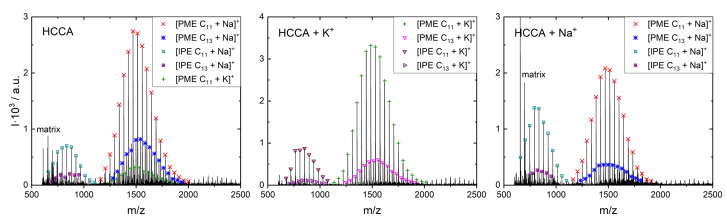
MALDI-TOF spectra of Tween20 from a pure HCCA matrix and from HCCA matrices upon addition of $K^{+}$ or ${\mathrm{Na}}^{+}$ salt.

**Figure A2:**
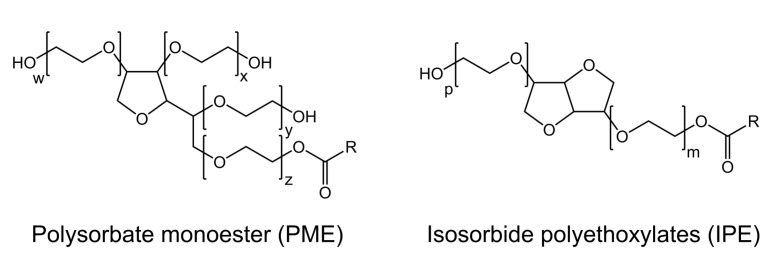
Molecular structures of the two major components found in commercially available Tween20: polysorbate monoester (PME) and isosorbide polyethoxylate (IPE). –R denotes the alkyl chain, –C_11_H_23_.

**Figure A3:**
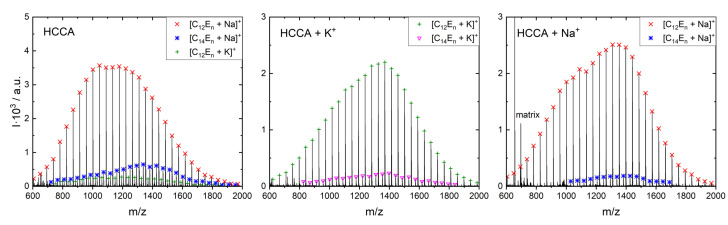
MALDI-TOF spectra of BrijL23 from a pure HCCA matrix and from HCCA matrices upon addition of $K^{+}$ or ${\mathrm{Na}}^{+}$ salt.

The nonionic surfactants Tween20 (also known as polysorbate 20, Figure 1a) and BrijL23 ($C_{12}E_{23}$, previous brand name: Brij 35, Figure 1b) were purchased from Sigma Aldrich (Darmstadt, Germany). The purity of both nonionic surfactants was analysed by mass spectrometry. The mass spectra of Tween20 (Figure A1) revealed a mixture composed of two major products: a polysorbate monoester and isosorbide polyethoxylate (Figure A2 and Table A1). The mass spectra of BrijL23 (Figure A3 and Table A2) showed only one major species: the alkyl poly-ethylene oxide ether as shown in Figure 1b. The anionic surfactant sodium dodecyl sulfate (SDS, ultrapure, Figure 1c) was purchased from PanReac AppliChem (Darmstadt, Germany). Heavy water ($D_{2}O$, 99.9 atom% D) was purchased from Sigma Aldrich (Darmstadt, Germany). All chemicals were used without further purification.

Before use, all glassware was cleaned by soaking in aqueous Hellmanex III (Hellma Analytics, Müllheim, Germany) solution for at least one hour and rinsing with large amounts of ultrapure water (milliQ-grade, 18.2 MΩ cm resistivity, Merck, Darmstadt, Germany). The surfactant solutions were prepared in $D_{2}O$. The mixed surfactant systems were prepared by mixing surfactant stock solutions. All samples were prepared three days before each experiment to allow sufficient dissolution. Detailed information on the surfactant properties is listed in Table A3.

### 2.2. Small-Angle Neutron Scattering (SANS)

Small-angle neutron scattering experiments were carried out on the D11 beamline at the Institut Laue-Langevin (ILL, Grenoble, France) [32]. A neutron wavelength of λ = 5.5 nm with Δλ/λ = 0.1 and sample-detector distances of 1.8 and 8 m were used to cover a *q*-range of 0.006–0.395 Å${}^{-1}$. Samples were measured in Hellma quartz cells with a path-length of 2 mm. The temperature was adjusted to 20.0 ${}^{\circ }C$. The sample scattering was normalised with respect to incident intensity, transmission, sample thickness, acquisition time and background. The data were brought to absolute scale, using ultrapure water as secondary standard. Data reduction was done using the Lamp software on site of the ILL.

### 2.3. Data Analysis

The scattering intensity I(q) is a function of the scattering vector magnitude *q* in micellar dispersions and is modelled as follows:

(1) $$I\left(q\right)=n_{p}\;P\left(q\right)\;S^{\prime }\left(q\right)\;+\;B.$$

Its intensity and shape depend on the number density of surfactant micelles $n_{p}$. *B* is a constant background that takes incoherent scattering, mostly from hydrogen, into account. Analysis of the reduced SANS data was done with build-in functions in the SASView 5.0.3 software (www.sasview.org, accessed on 5 May 2021).

**Figure 2:**
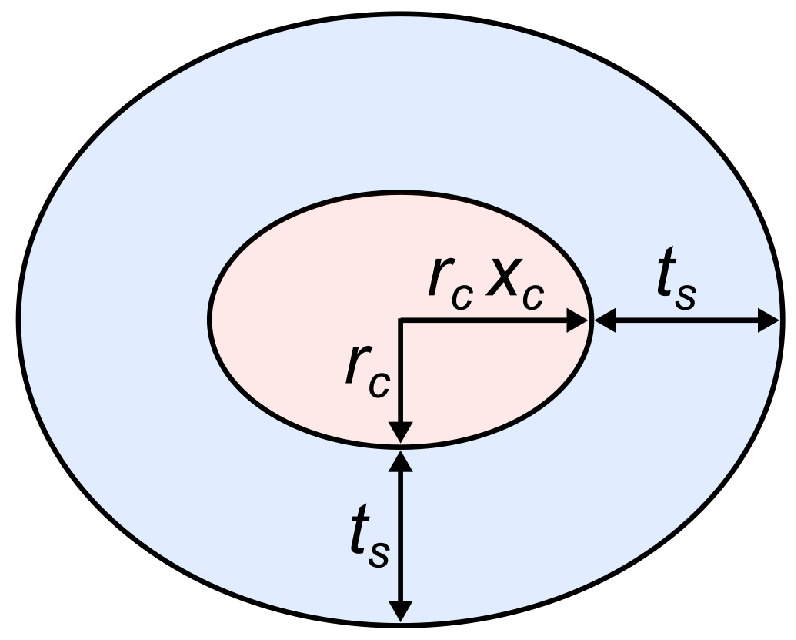
Schematic drawing of the core-shell ellipsoid model used for the calculation of the single particle form factor *P*(*q*).

A monodisperse core-shell ellipsoid model is used to describe the form factors P(q) of single micelles [33,34] (Figure 2, see SASView 5.0.3 documentation for details). The main parameters in this model are the equatorial radius of the core ($r_{c}$), the axial ratio between the polar and the equatorial radius of the core ($x_{c}$), and the thickness of the shell ($t_{s}$), which is assumed to be constant throughout the whole area. The scattering length density of the core ($\rho_{c}$), the shell ($\rho_{s}$), and the solvent ($\rho_{D_{2}O}$) account for the contrasts within the micelles.

A structure factor *S*(*q*) generally accounts for interparticle interactions and structure. In systems containing disordered ellipsoidal scatterers, an apparent, orientationally averaged interparticle structure factor $S^{\prime }\left(q\right)$ is approximated from the structure factor of isotropic scatterers, using the decoupling approximation. This approximation assumes that the interactions between the particles are independent from particle size and orientation, and are, therefore, only valid for small polydispersities and small anisotropies [33].

(2) $$S^{\prime }\left(q\right)=1+\frac{P{\left(q\right)}^{2}}{\langle |P\left(q\right){|}^{2}\rangle }\left(S\left(q\right)-1\right)$$

We use two different interaction potentials to fit the intermicelle structure factor S(q): for micelles with a charged surface, the RMSA (rescaled mean spherical approximation) [35] based on the mean spherical approximation (MSA) from Hayter and Penfold [36] is used. It describes the intermicellar interaction as a hard-sphere with a screened Coulombic potential. For uncharged particles, a hard-sphere interaction potential with the Percus–Yevick closure relationship is used (HS-PY) [37]. The RMSA and HS-PY closure relationship provides analytical solutions to the Ornstein–Zernike integral equations [38,39]. Both yield identical results in the case of hard-sphere fluids and can, therefore, be used in the transient regime between uncharged and charged micelles.

In this analysis, the volume fraction of micelles ϕ results from fitting the structure factor *S*(*q*), assuming the ellipsoidal micelles to occupy its equivalent volume of a sphere. The surface charge *z* per micelles is also fitted to *S*(*q*). No surface charge is extracted when the structure factor is modelled, using the hard-sphere interaction of uncharged particles. An effective micellar radius $r_{\mathrm{eff}}$ can be calculated for the prolate ellipsoidal micelles as follows:

(3) $$r_{\mathrm{eff}}={\left[{\left(r_{c}+t_{s}\right)}^{2}\left(r_{c}x_{c}+t_{s}\right)\right]}^{1/3}.$$

The aggregation number $N_{\mathrm{agg}}$ is calculated from the volume fraction of micelles ϕ, the effective radius $r_{\mathrm{eff}}$, the total surfactant concentration *c*, the critical micelle concentration (cmc), and Avogadros number $N_{A}$ as follows:

(4) $$N_{\mathrm{agg}}=\frac{4}{3}\pi r_{\mathrm{eff}}^{3}\frac{\left(c-cmc\right)N_{A}}{\phi }.$$

Finally, the micelles fractional charge β is defined for charged micelles as follows:

(5) $$\beta =\frac{z}{N_{\mathrm{agg}}}.$$

In order to reduce the number of fit parameters, the following assumptions are made: the micelle core consists of hydrophobic hydrocarbon chains; the shell contains the surfactant polar headgroups hydrated by water molecules that can penetrate the shell but not the core. The equatorial radius of the micelle core $r_{c}$ is fixed to 1.67 nm, being the length of a fully extended dodecyl hydrocarbon chain [40]. The core extends in the direction of the polar radius and is fitted by the axial ratio of the core $x_{c}$. We assume the hydrophobic micelle core to only contain hydrocarbon chains, with a fixed scattering length density (SLD) of $\rho_{c}$ = − 0.39 × 10${}^{-6}$ Å${}^{-2}$ (see Table A4). The solvent SLD is fixed to $\rho_{D_{2}O}$ = 6.34 × 10${}^{-6}$ Å${}^{-2}$. The fitted shell thickness $t_{s}$ is constant throughout the whole area of the micelle. The strong hydration of the surfactant polar groups is taken into account by fitting the SLD of the shell $\rho_{s}$. Self-consistent fitting is checked, using material balance equations from known molecular volumes and SLDs (further details in Table A4). Nonionic surfactants feature low critical micelle concentrations (cmc = 0.049 mM for Tween20 [41]; 0.09 mM for BrijL23 [9]) which are neglected in the calculations.

## 3. Results

### 3.1. Pure Micelles

**Figure 3:**
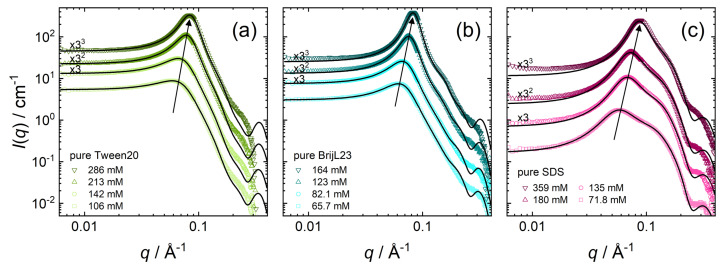
SANS data for micellar dispersions at various concentrations: (**a**) pure Tween20, (**b**) pure BrijL23, and (**c**) pure SDS. Symbols are experimental scattering data. The black solid lines are model fits to the data by the data by Equation (1). Data sets are scaled by the factors in black for clarity.

Small-angle neutron scattering (SANS) data of pure surfactant solutions are shown in Figure 3 for the three different surfactants used in this study: Tween20 (Figure 3a), BrijL23 (Figure 3b), and SDS (Figure 3c). These systems are measured at high surfactant concentrations at which a pronounced intermicellar structuring is expected.

SANS data in Figure 3 are quantitatively analysed by a core-shell ellipsoid model; the theoretical background on the analysis of SANS data can be found in Section 2.3. The black solid lines display the model fits to the corresponding data sets, according to Equation (1). Overall, the model fits and the experimental data points are in excellent agreement except at high *q*-values. The small distances that are probed at high *q*-values (e.g., molecular inhomogeneities between water and surfactant headgroups in the shell) are not described by the simple core-shell model assuming homogeneous scattering from cores and shells. Therefore, deviation between fit and data points at high *q*-values is expected.

#### 3.1.1. Pure Nonionic Micelles

SANS data of pure Tween20 (Figure 3a) show a stronger structure factor peak with increasing Tween20 concentration, denoting stronger intermicellar interactions at higher surfactant concentrations. Apart from the peak intensity, the peak position shifts toward higher *q*-values (see arrow in Figure 3a) and with smaller intermicellar distances at higher Tween20 concentrations. The scattering curves of Tween20 (with its branched headgroup) are similar to the ones of the linear and well-known nonionic BrijL23 surfactants (Figure 3b), indicating a similar shape and type of interactions of the pure nonionic micelles. The concentration dependence of the structure peak of BrijL23 follows the same trends as for Tween20.

Table 1 summarises the parameters extracted from the SANS model fits (core-shell ellipsoid + HS-PY structure factor) of both pure nonionic surfactants: Tween20 and BrijL23, with *c* being the surfactant concentration. The fitted values are as follows: volume fraction ϕ, axial ratio of the core $x_{c}$, shell thickness $t_{s}$, and the shell scattering length density $\rho_{s}$. The calculated values are as follows: the effective radius $r_{\mathrm{eff}}$ (Equation (3)), and the aggregation number $N_{\mathrm{agg}}$ (Equation (4)).

The core axial ratios $x_{c}$ of 1.72–2.21 indicate the formation of prolate ellipsoids in all cases. The thicknesses of the hydrated shells $t_{s}$ are 1.72–1.81 nm for Tween20 micelles. At higher Tween20 concentrations, the micelle size decreases slightly, from an effective radius $r_{\mathrm{eff}}$ of 4.05 nm at 106 mM to 3.75 nm at 286 mM. In general, pure Tween20 micelles comprise smaller micelles, compared to the pure BrijL23 micelles. The effective radii $r_{\mathrm{eff}}$ of pure BrijL23 micelles range from 4.28–4.02 nm. The aggregation numbers $N_{\mathrm{agg}}$ of Tween20 are with values of 92–102 higher than for pure BrijL23 micelles, which contain 58–61 surfactants per micelle. Pure BrijL23 micelles are less elliptical, compared to pure Tween20 micelles, with core axial ratios $x_{c}$ of 1.69–1.89.

#### 3.1.2. Pure SDS Micelles

In order to fit the SANS data measured for pure SDS (Figure 3c) some assumptions made for pure nonionic micelles have to be adjusted. The critical micelle concentration cmc for anionic surfactants is substantially higher than for nonionic surfactants and, therefore, cannot be neglected in the calculations. The cmc depends on the total SDS surfactant concentration. The cmc is estimated from activity measurements [42,43] for each SDS concentration separately. SDS micelles are assumed to posses a strongly bound solvent hydration shell with a constant thickness $t_{s}$ of 0.69 nm [44,45]. The scattering length density (SLD) of the hydration shell corresponds to the solvent SLD of $\rho_{s}$ = 6.34 × 10${}^{-6}$ Å${}^{-2}$ (Table A4). Additional scattering of the sulfate headgroup (with associated sodium counterions) is neglected because their volume fraction in the hydration shell is ≲0.1, while its SLD of 5.2 × 10${}^{-6}$ Å${}^{-2}$ is close to the SLD of the solvent [45]. Consequently, the headgroup SLD is in good approximation, defined by the solvent SLD only. The volume fraction ϕ is then calculated from the volume ratio of the micelles, including their hydration shell, the core volume, the known molecular volume $v_{m}$ of the hydrophobic dodecyl chain (see Table A4), the Avogadro number $N_{A}$, the SDS concentration *c* and the cmc as follows:

(6) $$\phi =\frac{\left(x_{c}r_{c}+t_{s}\right){\left(r_{c}+t_{s}\right)}^{2}}{x_{c}r_{c}^{3}}v_{m}N_{A}\left(c-cmc\right)$$

Table 2 summarises the parameters extracted from the SANS model fits (core-shell ellipsoid + RMSA structure factor) of pure SDS micelles. *c* is the surfactant concentration. The fitted values are as follows: axial ratio of the core $x_{c}$ and the charge per micelles *z*. The calculated values are as follows: volume fraction ϕ (Equation (6)), the effective radius $r_{\mathrm{eff}}$ (Equation (3)), and the aggregation number $N_{\mathrm{agg}}$ (Equation (4)).

Overall, the fit parameters are in good agreement with the literature values [46,47,48,49]. The trend of increasing aggregation number $N_{\mathrm{agg}}$ with its concentration is well known for SDS [44,50].

### 3.2. Mixed Nonionic–Anionic Micelles

**Figure 4:**
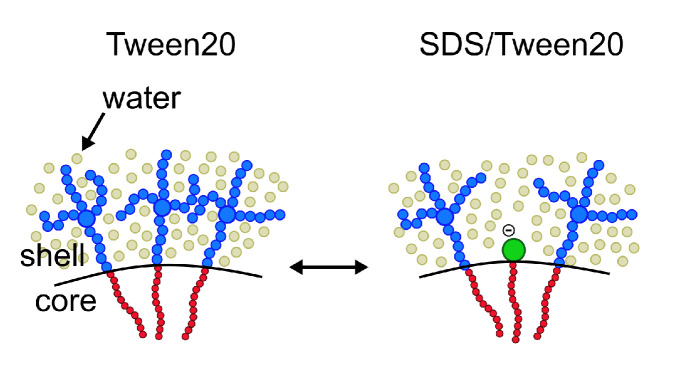
Surfactants are assumed to homogeneously distribute and form mixed nonionic–anionic micelles, as visualised for SDS/Tween20 mixtures.

After describing pure nonionic and anionic micelles, the effect of mixing nonionic surfactants (Tween20 or BrijL23) and anionic SDS surfactants is investigated. Ideally, both surfactant species distribute homogeneously and form mixed nonionic–anionic micelles (visualised for SDS/Tween20 mixtures in Figure 4).

#### 3.2.1. Mixed Micelles in Dilute Conditions

**Figure 5:**
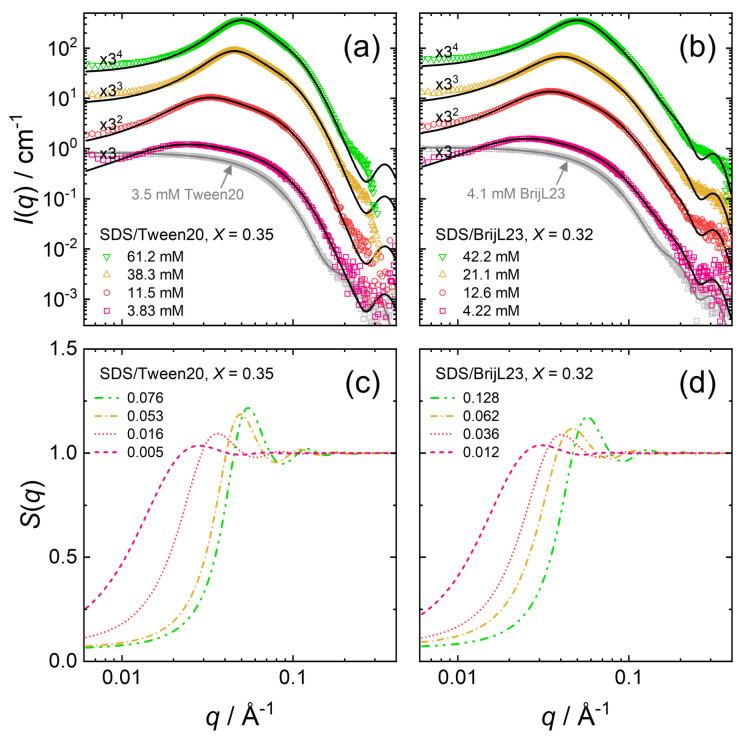
SANS data for mixed micellar dispersions at various concentrations: (**a**) SDS/Tween20, (**b**) SDS/BrijL23. Symbols are experimental scattering data. The black solid lines are model fits to the data by Equation (1). Data sets are scaled by the factors in black for clarity. Extracted structure factors S(q) from the model fits: (**c**) SDS/Tween20, (**d**) SDS/BrijL23. Data are given for different volume fractions ϕ.

Figure 5 shows the SANS scattering data of mixed solutions of (a) nonionic Tween20 and anionic SDS and (b) nonionic BrijL23 and anionic SDS. The molar mixing ratio *X* = [SDS]/([SDS] + [nonionic]) is kept constant at *X* = 0.35 and *X* = 0.32, for SDS/Tween20 and SDS/BrijL23, respectively. The total surfactant concentration *c*, [SDS] + [nonionic], is varied. For comparison, the scattering data of the respective pure nonionic surfactant solutions are added to the graph (grey squares).

The concentration of the pure nonionic surfactant solutions (3.5 mM Tween20 or 4.1 mM BrijL23) is well above the surfactant’s cmc, and self-assembled micelles are formed. The uncharged micelles are diluted and no intermicellar structuring occurs. The scattering curves are, therefore, fitted using only the core-shell form factor *P*(*q*) (with the structure factor *S*(*q*) = 1). The scattering data SDS/Tween20 (*X* = 0.35) and SDS/BrijL23 (*X* = 0.32) reveal that mixed nonionic–anionic micelles form in both cases. Both types of surfactants self-assemble into mixed micelles already at total surfactant concentrations below the cmc of pure SDS (8.1 mM, see Table A3). Nonionic micelles act as a “seed” and the SDS surfactant molecules incorporate. In contrast to the pure nonionic micelles, mixed micelles show intermicellar structuring, due to long-ranged electrostatic interactions already at small volume fractions (ϕ ≈ 0.005). The intermicellar structuring strengthens with increasing volume fraction ϕ as indicated by a more pronounced structure factor *S*(*q*) (Figure 5c,d).

Table 3 summarises the parameters extracted from the SANS model fits (core-shell ellipsoid + RMSA structure factor) of SDS/Tween20 and SDS/BrijL23 solutions. The fitted values are as follows: volume fraction ϕ, axial ratio of the core $x_{c}$, shell thickness $t_{s}$, the shell scattering length density $\rho_{s}$, and the charge per micelle *z*. The calculated values are as follows: the effective radius $r_{\mathrm{eff}}$ (Equation (3)), the aggregation number $N_{\mathrm{agg}}$ (Equation (4)), and the fractional charge β (Equation (5)).

The mixed micelles adapt similar morphologies (size and shape) as the pure nonionic surfactant micelles. Mixed micelles based on Tween20 (with its branched PEO headgroup) comprise smaller micelles, compared with the mixed micelles based on BrijL23 (with its linear PEO headgroup) as nonionic surfactants.

#### 3.2.2. Concentrated Mixed Micelles

**Figure 6:**
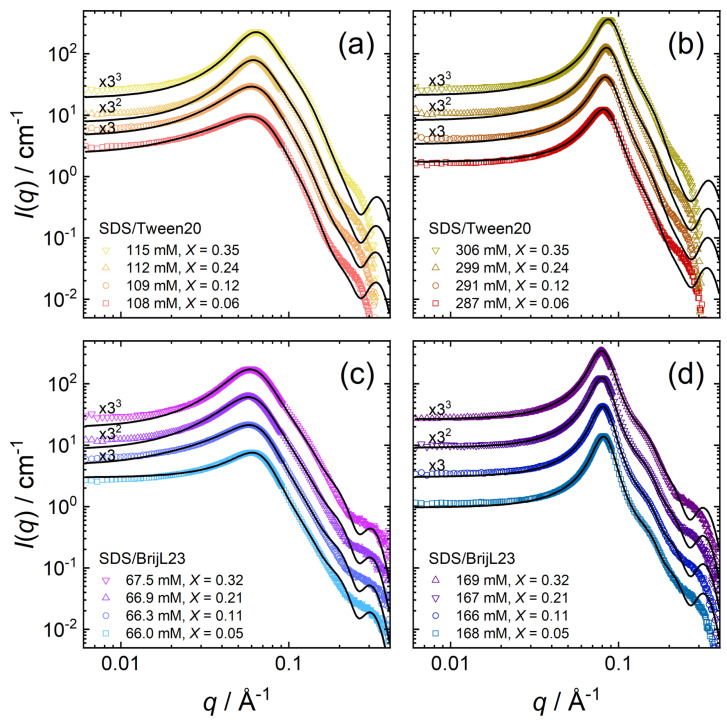
SANS data for surfactant mixtures at varying mixing ratios *X* = [SDS]/([SDS] + [nonionic]): (**a**,**b**) SDS/Tween20, and (**c**,**d**) SDS/BrijL23. Symbols are experimental scattering data. The black solid lines are model fits to the data by Equation (1). Data sets are scaled by the factors in black for clarity.

Figure 6 shows SANS data of mixed surfactant solutions at high total surfactant concentrations *c* and at various mixing ratios *X*.

SANS data in Figure 6a,c clearly reveal the effect of the substitution of nonionic with anionic surfactants on intermicellar structuring. With increasing *X*, i.e., at higher amounts of anionic surfactant, the structure factor peak intensifies, while the general shape of the scattering curves remains similar. The low impact on the curve shape confirms no significant architecture (i.e., form factor) modifications of the single micelles upon admixing SDS. The peak position shifts towards lower *q*-values at higher mixing ratios *X*. Interestingly, at higher surfactant concentrations and, therefore, at higher volume fractions ϕ, a higher SDS proportion has less influence on the structure factor in Figure 6b,d. Not only does the peak position remain constant, but also the peak intensity and peak width are only slightly affected. At small mixing ratios *X* and at high micellar volume fractions, the intermicellar interactions cannot be fitted using the RMSA structure factor because of the high uncertainty in the micellar charge *z*. In those cases, fitting is carried out using the HS-PY structure factor, assuming uncharged micelles. The applicability of this procedure will be discussed in Section 4.2.

Table 4 and Table 5 summarise the parameters extracted from the SANS model fits (core-shell ellipsoid + RMSA structure factor) of SDS/Tween20 and SDS/BrijL23 solutions, respectively. The fitted values are as follows: volume fraction ϕ, axial ratio of the core $x_{c}$, shell thickness $t_{s}$, shell scattering length density $\rho_{s}$, and the charge per micelle *z*. The calculated values are as follows: the effective radius $r_{\mathrm{eff}}$ (Equation (3)), the aggregation number $N_{\mathrm{agg}}$ (Equation (4)), and the fractional charge β (Equation (5)).

At all mixing ratios *X* investigated, the nonionic–anionic micelles can be modelled as core-shell ellipsoids with only little variation in micelle morphology. The effective radii $r_{\mathrm{eff}}$ of all SDS/Tween20 micelles are in the range of 4.10–3.77 nm. Mixed micelles of SDS/BrijL23 are larger than mixed micelles from SDS/Tween20, having effective radii in the range of 4.44–4.05 nm. Similar to the pure nonionic micelles, mixed micelles of SDS/BrijL23 form larger micelles, although their aggregation number is smaller compared to mixed micelles of SDS/Tween20.

#### 3.2.3. Non Ideal Behaviour of Mixed Nonionic–Anionic Micelles

**Figure 7:**
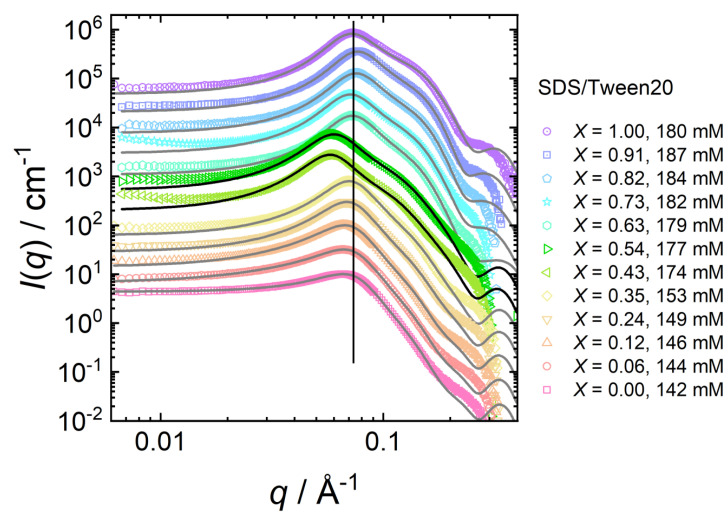
SANS data for mixed Tween20-SDS surfactant mixtures at similar surfactant concentrations but at varying mixing ratios *X* = [SDS]/([SDS] + [Tween20]). Symbols are experimental scattering data. The black solid lines are model fits to the data by Equation (1). Data sets are scaled with a factor of $3^{n}$ for clarity.

Figure 7 shows SANS data of mixed SDS/Tween20 micelles at various mixing ratios *X*, starting from *X* = 0.00 (pure Tween20) to *X* = 1.00 (pure SDS) with similar total surfactant concentrations (142–187 mM).

While the micellar size and shape remain stable up to a SDS molar ratio of *X* = 0.35 (Section 3.2.2), they change at intermediate mixing ratios (*X* = 0.43, 0.54) as indicated by the pronounced shift in the structure factor peak. At even higher SDS molar ratios (*X* ≥ 0.64), all scattering curves recorded for ratios up to pure SDS micelles look similar.

Table 6 summarises the parameters extracted from the SANS model fits (core-shell ellipsoid + RMSA structure factor; pure Tween20: HS-PY structure factor) of SDS/Tween20 solutions. The fitted values are as follows: volume fraction ϕ, axial ratio of the core $x_{c}$, shell thickness $t_{s}$, shell scattering length density $\rho_{s}$, and the charge per micelle *z*. The calculated values are as follows: the effective radius $r_{\mathrm{eff}}$ (Equation (3)), the aggregation number $N_{\mathrm{agg}}$ (Equation (4)), and the fractional charge β (Equation (5)).

**Figure 8:**
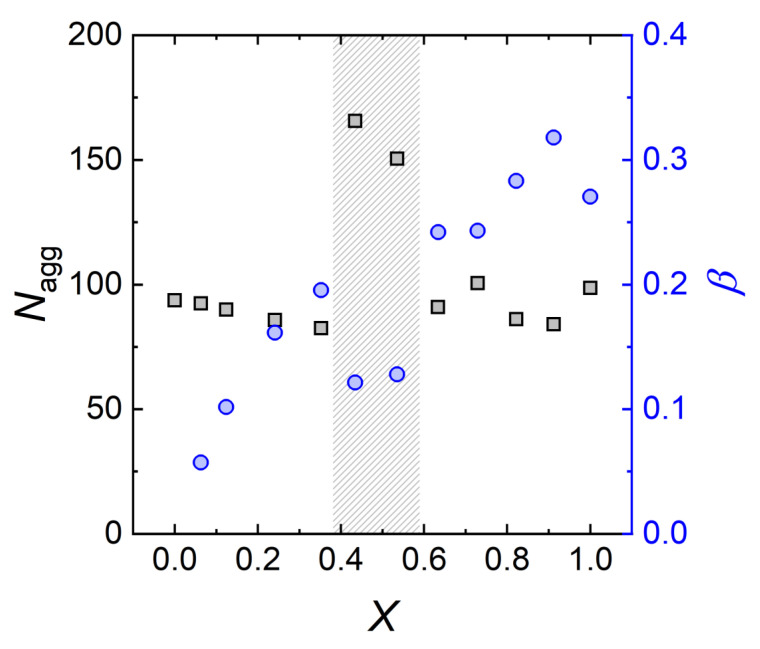
Aggregation number $N_{\mathrm{agg}}$ and fraction charge β of the formed mixed SDS/Tween20 micelles determined by the fits of SANS data depending on the SDS molar ratio *X*.

The evolution of the aggregation number $N_{\mathrm{agg}}$ and the fractional charge β of the formed mixed SDS/Tween20 micelles are shown in Figure 8 in dependence of the mixing ratio *X* for better visualisation of both parameters.

Figure 8 clearly shows the non-linear behaviour of the mixed micelles at intermediate SDS molar ratios (*X* ≈ 0.4–0.6). Up to *X* = 0.35, the aggregation number $N_{\mathrm{agg}}$ (black squares) is almost constant, while the fractional charge β (blue circles) indicates a continuous introduction of surface charges onto the micelles. At SDS molar ratios of *X* ≈ 0.4–0.6, the micellar size and shape change drastically. The aggregation number peaks to 150–166 while the fractional charge drops significantly. At even higher SDS molar ratios, the aggregation number reduces again to values of 84–100, while the fractional charge of the micelles approaches a value of ≈0.3, continuing to follow the initial trend.

## 4. Discussion

### 4.1. Micelle Formation

#### 4.1.1. Nonionic Surfactants with Branched or Linear Headgroups

Self-assembled structures of Tween20 are already reported in the literature either as globular core-shell micelles [51] or as core-shell ellipsoids [52,53]. A recent study showed that hard-sphere interactions adequately describe the interactions between pure Tween20 micelles [54]. At higher degrees of ethoxylation (>20), partial charges on the ether oxygen in the EO groups enhance the intermicellar interactions. The literature reports on different properties of pure Tween20 micelles with aggregation numbers ranging from 349 [51], 90 [52], 70 [55], to 22 [53] accompanied by varying micelle dimensions. Our results are in good agreement with the results obtained by Penfold et al. [52]. The aggregation numbers and core radii are similar, while only the shell thickness is around ≈0.5 nm thicker. The difference probably results from the purity of the Tween20 used. In our work, we used commercially available Tween20, whereas the surfactants in the study of Penfold et al. were specifically synthesised. The Tween20 used in this work was analysed by mass spectrometry, identifying two major products (Figure A2). Both products have predominantly $C_{11}$ alkyl chains but different distributions of head groups: one head group contains 26, while the other one contains 11 EO groups on average. The surfactant mixture contains 20 EO groups, on average, as indicated by the manufacturer (details in Table A1). The presence of larger head groups located in the shell might explain the thicker shell when using the commercial product.

Nonionic BrijL23 surfactants consist of a linear poly-ethylene oxide (PEO) chain as the headgroup. Their self-assembly was also already studied, using light scattering [56], small-angle neutron scattering [57,58], small-angle X-ray scattering [59] and Monte Carlo simulations [60]. In this study, we used a core-shell ellipsoid form factor and a structure factor, assuming the micelles to interact as hard-spheres. Recent studies used different form factors, such as tethered spheres [60] as well as the more sophisticated intermicellar interaction potential, such as the soft-sphere core potential [57]. Our model fits describe the data very well, and the fit parameters remain in good agreement with the literature values.

Interestingly, BrijL23 surfactant molecules self-assemble into larger micelles compared to Tween20, although BrijL23 micelles comprise a lower aggregation number. The linear BrijL23 headgroups are more hydrated with the solvent, compared to the branched headgroups of Tween20. This reflects in the higher scattering length density of the shells from BrijL23 micelles, due to the increased proportion of $D_{2}O$. Material balance equations from known molecular volumes and SLDs reveal a slight mismatch of core volumes if only comprised of hydrocarbon chains and the SLD of the shell, assuming homogeneous scattering for both nonionic micelles. An inhomogeneous distribution of EO-groups within the micelles may describe this phenomenon. Some EO groups may enter the hydrophobic core and accumulate at the core-shell interface. This was already demonstrated for $C_{12}E_{6}$ surfactants using molecular dynamics simulations [61], or for $C_{18}E_{100}$ using SANS [62].

#### 4.1.2. On the Nature of Mixed Micelles with Nonionic (PEO) and Anionic (Sulfate) Headgroups

When mixing nonionic PEO-based surfactant molecules with anionic SDS surfactant molecules, mixed micelles form. At higher SDS ratios, formation of smaller mixed micelles is expected because the volume of a sulfate headgroup is around 30 times smaller, compared to the nonionic PEO headgroups. The smaller headgroup volume of SDS, however, is almost completely compensated by the increased hydration of the shell at higher SDS ratios. Hydration of the headgroup does not only affect the size of the micelle, but also the contrast of the shell and with that, its scattering length density (SLD).

The distribution of charged sites across the micelles surface indicates that both types of surfactants (nonionic–anionic) are homogeneously distributed in the mixed micelles formed. A homogeneous distribution might not only be favoured by the hydrophobic effect of the alkyl chains, but also by favourable interactions between the EO and sulfate headgroups. This is assumed since favourable interactions between EO and sulfate groups were previously reported for linear PEO polymers and SDS surfactants dissolved in water. They revealed that strong polymer–surfactant complexes form [63,64,65,66,67]. These interactions seem to exclusively occur in the intramicellar structure since it is not necessary to introduce attractive interactions to fit the intermicellar structure factor, even when the micelles are highly concentrated.

The counterion condensation plays an important role on the micellar surface charge. Although the charge per micelle *z* increases with increased mixing ratio *X*, the strength of counterion binding to the micelles varies. This is emphasised on the SDS/Tween20 mixtures: at a mixing ratio of *X* = 0.12, the fractional charge of the micelles is β = 0.09–0.11. The similarity of both values indicates that most counterions are dissociated from the sulfate headgroup. Exact calculations (degree of SDS dissociation = β/*X*, assuming all SDS molecules to incorporate into the micelles) show that at *X* = 0.12, between 78 and 92% of the SDS headgroups are dissociated. At *X* = 0.35, the counterion condensation elevates and only between 44 and 59% of SDS headgroups remain dissociated. Thus, the surface charge and the mixing ratio do not linearly depend on each other. Electrostatic limitations, similar to the Manning limiting law [68], influence the effective surface charge. The role of the counterion condensation is well known from studies on other mixed micelles, such as from nonionic $C_{12}E_{23}$ surfactants with SDS [69], or from nonionic, sugar-based surfactants with SDS [47].

In Section 3.2.3, we explored the non-ideal behaviour when mixing nonionic Tween20 and anionic SDS surfactants. Similar behaviour was also found in mixed SDS/$C_{12}E_{6}$ surfactant solutions [28]: for a specific surfactant molar ratio, the aggregation number exhibits a pronounced maximum together with a significant micellar growth. The intermolecular interactions between PEO and sulfate headgroups are described as a balance of steric and electrostatic contributions [17,70,71]. Upon addition of SDS, the micelles grow in size due to the reduction of the steric interactions between the $C_{12}E_{6}$ headgroups. At high SDS concentrations, however, electrostatic repulsion between the sulfate groups increases the aggregates curvature and smaller micelles are formed again. In our study, non-linear micelle growth is found at an SDS molar ratio of *X* ≈ 0.5. Comparing the ratio between EO and sulfate groups, with the study of Penfold et al. [28] reveals that in both cases this behaviour occurs at the same sulfate/EO ratio of ≈0.04–0.05, i.e., roughly one sulfate group per 20 EO groups. These two studies suggest that only the ratio between EO- and sulfate groups matter. Of interest would be to study the behaviour of very short, e.g., $C_{12}E_{1}$, or very long, e.g., $C_{12}E_{100}$, nonionic surfactants mixed with SDS to further explore the influence of the steric contribution.

### 4.2. Intermicellar Structuring at Various Volume Fractions

**Figure 9:**
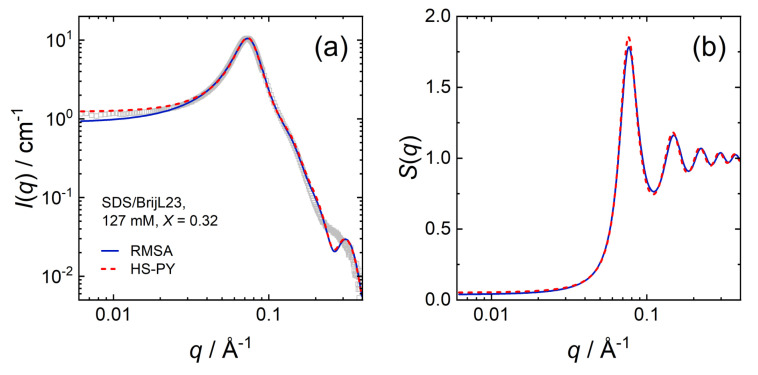
(**a**) SANS data for the SDS/BrijL23 mixture with a total surfactant concentration of 127 mM and mixing ratios *X* = [SDS]/([SDS] + [BrijL23]) = 0.32. Symbols are experimental scattering data. Two model fits to the data by Equation (1) are compared: the blue, solid line is fitted using the RMSA structure factor. The red, dashed line is the result, using the HS-PY structure factor. Extracted structure factors *S*(*q*) are displayed in panel (**b**).

Some results in Section 3.2.2 suggest that the intermicellar structuring of charged micelles may be well described treating the micelles as uncharged hard-spheres once they are highly concentrated. At large volume fractions of dispersed micelles, the excluded volume effect is the dominant driving force for the intermicellar structure. The scattering data of mixed SDS/BrijL23 micellar dispersions, with a mixing ratio of *X* = 0.32 and a total surfactant concentration of 127 mM was fitted with both the RMSA and the HS-PY structure factor *S*(*q*) for comparison. Figure 9 shows that the results from both methods agree well with each other.

Table 7 summarises the parameters extracted from the SANS model fits (core-shell ellipsoid + both structure factors). The fitted values are as follows: volume fraction ϕ, axial ratio of the core $x_{c}$, shell thickness $t_{s}$, shell scattering length density $\rho_{s}$, and the charge per micelle *z*. The calculated values are as follows: the effective radius $r_{\mathrm{eff}}$ (Equation (3)), the aggregation number $N_{\mathrm{agg}}$ (Equation (4)), and the fractional charge β (Equation (5)).

Both approaches result in similar fit parameters for the intra- and intermicellar structuring, with the difference that no surface charges are extracted when using the HS-PY structure factor. The long range electrostatic interactions between charged micelles become more dominant in the description of intermicellar structuring when the micelles are more diluted and, therefore, more separated.

The mean intermicellar distance in the dispersion is evaluated from the respective structure factors *S*(*q*). The extracted structure factor *S*(*q*) of micelles with near range ordering shows a pronounced first-order peak that can be fitted to a Lorentzian profile as follows:

(7) $$S\left(q\right)=\frac{S_{\max}{\left(\frac{\Delta q}{2}\right)}^{2}}{{\left(q-q_{\max}\right)}^{2}+{\left(\frac{\Delta q}{2}\right)}^{2}}+S_{0}$$

where $S_{\max}$ denotes the intensity of the peak, Δq is the full width at half maximum (FWHM), $q_{\max}$ the position and $S_{0}$ the baseline of the peak. The Lorentzian peak profile is the Fourier transformation of the radial distribution function *g*(*r*) of the micelles. Because of this, the Lorentzian lineshape of *S*(*q*) is used for the description of the micellar bulk structuring [72,73,74].

The mean intermicellar distance D* is calculated from the position of the peak maximum $q_{\max}$ as D* = $\frac{2\pi }{q_{\max}}$. The inverse cubic root law estimates the mean intermicellar distances D* for charged particles (Equation (8)), considering simple packing arguments, knowing the effective particle radius $r_{\mathrm{eff}}$ and the particle volume faction ϕ.

(8) $$D*=f\;r_{\mathrm{eff}}\;\phi^{-1/3}$$

**Figure 10:**
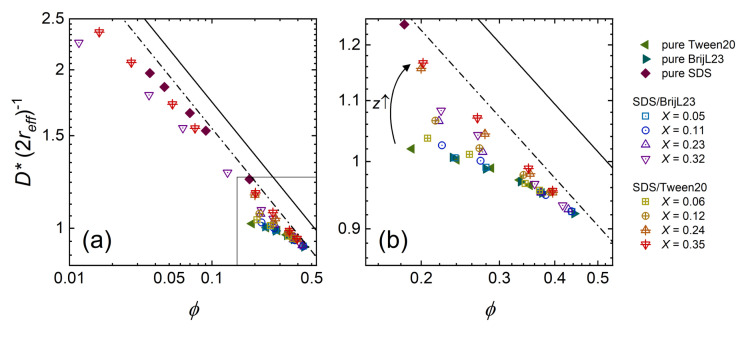
The mean intermicellar distance D* normalised to the effective micelle diameter (2$r_{\mathrm{eff}}$) in dependency of the volume fraction ϕ for all systems investigated. The lines are predictions according to Equation (8) with a pre-factor of *f* = 1.436 (dashed line) and *f* = 1.612 (solid line). Panel (b) is a magnification of the box in panel (**a**). The arrow in panel (**b**) highlights the influence of a higher surface charge *z*.

The type of particle packing determines the value of the pre-factor *f*. For a simple cubic packing D* = ${\left(4/3\pi \right)}^{1/3}r_{\mathrm{eff}}\;\phi^{-1/3}$, so that *f* = 1.612. Experimentally, *f* = 1.436 is found for microemulsions [75]. In Figure 10, the bulk intermicellar distance D* is normalised to the effective diameter (2$r_{\mathrm{eff}}$).

Both types of pure nonionic micelles (BrijL23 and Tween20) follow the same volume fraction dependency. The intermicellar distance varies only very little with the volume fraction ϕ. At higher ϕ, the values of nonionic micelles approach the scaling behaviour for charged particles. This is expected since at close packing geometries (ϕ ≈ 0.52–0.74) the interparticle distances of charged and uncharged particles do not differ.

In the same way, it is reasonable that the mean distance D* between micelles at ϕ ≈ 0.35 changes only slightly by introducing surface charges to the micelles. At ϕ ≈ 0.20, the intermicellar distance subsequently increases with increasing the micellar surface charge *z*. The electrostatic repulsion between micelles increases with the amount of surface charges. As a result, the micelles arrange in a preferred order, which is maintained towards smaller volume fractions (ϕ ≈ 0.01). When the micelles exceed a certain amount of surface charge—in this study, mixed micelles at high mixing ratio *X* as well as pure SDS micelles—they follow approximately the inverse cubic root scaling law proposed for charged particles (Equation (8) with *f* = 1.436, dashed line in Figure 10).

At high volume fractions (ϕ > 0.3), the intermicellar distance is smaller than the effective diameter of the micelle as indicated by a value below 1 of D* ($2r_{\mathrm{eff}}$)${}^{-1}$. The effective radius is calculated, assuming the ellipsoid to occupy the same volume as a sphere. A closer intermicellar distance than the effective diameter may result from a preferred alignment of the ellipsoids. The micelles’ ellipticity reaches a total aspect ratio of 1.58, due to the elliptical hydrophobic core but a constant shell thickness. However, no explicit anisotropy is observed in the scattering detector image. Furthermore, the apparent structure factor $S^{\prime }$(*q*) determined by the decoupling approximation may comprise some inaccuracies at volume fractions and ellipticities used in this study [76].

## 5. Conclusions

We measured mixed nonionic–anionic surfactant solutions over a wide range of solution compositions and concentrations using SANS. For all cases, the self-assembled structures can be described as core-shell ellipsoidal micelles. The following conclusions can be drawn:

The formation of mixed nonionic–anionic (SDS) micelles takes place irrespective of whether the nonionic hydrophilic headgroup consists of branched PEO groups (Tween20) or a linear PEO chain (BrijL23). Mixed micelles with nonionic micelles containing linear PEO chains are typically larger and comprise a more hydrated shell, compared to the mixed micelles containing branched nonionic headgroups. Mixed micelles already form below the critical micelle concentration of pure SDS, showing the synergistic nature of the mixed micelle formation. The formation of mixed micelles may not only be favourable due to hydrophobic interactions, but also due to favourable interaction between the nonionic PEO and anionic sulfate headgroups. At almost all mixing ratios, the micellar size and shape is almost independent of the total surfactant concentration and the micelles may be concentrated to volume fractions of ϕ ≳ 0.45. At roughly equimolar mixing ratios (*X* ≈ 0.4–0.6), however, the micelles increase non-linearly in size and aggregation number, possibly due to a change in electrostatic and steric contributions to the free energy in micelle formation.

At high surfactant concentrations, i.e., at very large volume fractions of micelles, intermicellar structuring is completely dominated by excluded volume effects. Consequently, almost no difference in intermicellar structuring occurs between charged and uncharged micelles at volume fractions above ϕ ≈ 0.3. The mean intermicellar distance D* of charged micelles can be estimated by their volume fraction ϕ, according to D* $\propto \phi^{-1/3}$. At less or even uncharged micelles, this scaling dependency is no longer valid since the intermicellar structuring is less pronounced due to the absence of the long-ranged electrostatic repulsion.

In summary, this article provides an overview of mixed PEO-sulfate surfactant micelles and shows that their surface charge can gradually be tuned by changing the mixing ratio of nonionic and anionic surfactants while their aggregation number remains almost unchanged. Only at roughly equimolar ratios of nonionic and anionic surfactants do deviations in the self-assembly occur. The provided description of the intermicellar nanostructuring with respect to the micellar surface charge will be beneficial for the description of depletion effects in colloidal dispersions. This will be studied in detail by measuring oscillatory structural forces, which are present in thin films from micellar dispersions at high effective volume fractions.

## Figures and Tables

**Table 1:** Parameters extracted from SANS model fits (core-shell ellipsoid form factor + PY structure factor) of pure nonionic surfactants (Tween20 or BrijL23) in $D_{2}O$ at 20.0 ${}^{\circ }$C.

	*c*	ϕ	$x_{c}$	$t_{s}$	$\rho_{s}$·10${}^{-6}$	$r_{\mathrm{eff}}$	$N_{\mathrm{agg}}$
	mM			nm	Å${}^{-2}$	nm	
Tween20	106	0.190	2.21	1.81	5.06	4.05	94
	142	0.240	2.14	1.78	5.08	3.99	95
	213	0.333	1.93	1.72	5.10	3.85	92
	286	0.374	1.72	1.72	5.18	3.75	101
BrijL23	65.7	0.235	1.93	2.21	5.45	4.34	57
	82.1	0.280	1.89	2.17	5.42	4.28	58
	123	0.374	1.77	2.09	5.38	4.14	59
	164	0.442	1.69	2.00	5.35	4.02	61

**Table 2:** Parameters extracted from SANS model fits (core-shell ellipsoid form factor + RMSA structure factor) of pure anionic SDS in $D_{2}O$ at 20.0 ${}^{\circ }C$. The volume fraction ϕ and the effective radius $r_{\mathrm{eff}}$ refer to micelles, including their hydration shell.

	*c*	cmc	ϕ	$x_{c}$	$r_{\mathrm{eff}}$	$N_{\mathrm{agg}}$	β
	mM	mM			nm		
SDS	71.8	3.9	0.037	1.49	2.61	83	0.28
	135	2.8	0.070	1.64	2.67	91	0.29
	180	1.6	0.090	1.72	2.71	99	0.27
	359	1.0	0.183	1.95	2.80	108	0.25

**Table 3:** Parameters extracted from SANS model fits (core-shell ellipsoid form factor + RMSA structure factor) of mixed nonionic–anionic micelles in $D_{2}O$ at 20.0 ${}^{\circ }C$.

*X*	*c*	ϕ	$x_{c}$	$t_{s}$	$\rho_{s}$·10${}^{-6}$	$r_{\mathrm{eff}}$	$N_{\mathrm{agg}}$	β
	mM			nm	Å${}^{-2}$	nm		
SDS/Tween20								
0.35	3.83	0.005	1.94	1.85	5.35	3.70	94	0.10
	11.5	0.016	1.87	1.82	5.36	3.61	85	0.15
	38.3	0.053	1.88	1.74	5.40	3.63	88	0.18
	61.2	0.076	1.90	1.74	5.36	3.65	99	0.17
SDS/BrijL23								
0.32	4.22	0.012	1.89	2.28	5.78	4.39	77	0.12
	12.7	0.036	1.80	2.20	5.71	4.28	69	0.17
	21.1	0.062	1.82	2.18	5.68	4.26	66	0.18
	42.2	0.128	1.77	2.18	5.64	4.24	63	0.20

**Table 4:** Parameters extracted from SANS model fits (core-shell ellipsoid form factor + RMSA structure factor) of mixed SDS/Tween20 surfactants in $D_{2}O$ at 20.0 ${}^{\circ }C$. For some solutions, fits with the RMSA structure factor do not converge and fitting is carried out using the HS-PY structure factor instead with no fractional charge β extracted.

*X*	*c*	ϕ	$x_{c}$	$t_{s}$	$\rho_{s}$·10${}^{-6}$	$r_{\mathrm{eff}}$	$N_{\mathrm{agg}}$	β
	mM			nm	Å${}^{-2}$	nm		
0.06	108	0.207	2.26	1.83	5.14	4.10	91	0.06
	143	0.257	2.17	1.80	5.14	4.03	92	0.06
	216	0.343	2.09	1.77	5.21	3.96	98	–
	287	0.369	1.85	1.73	5.26	3.82	109	–
0.12	109	0.215	2.21	1.85	5.22	4.10	88	0.11
	146	0.270	2.17	1.82	5.21	4.05	90	0.10
	218	0.340	2.01	1.74	5.20	3.90	96	0.09
	291	0.390	1.85	1.67	5.19	3.76	100	0.08
0.24	112	0.200	2.02	1.78	5.37	3.94	87	0.18
	149	0.278	2.12	1.78	5.33	3.99	86	0.16
	224	0.352	1.99	1.71	5.30	3.86	93	0.13
	299	0.396	1.87	1.63	5.28	3.73	99	0.11
0.35	115	0.202	1.92	1.71	5.44	3.83	81	0.21
	153	0.268	1.96	1.71	5.47	3.85	82	0.20
	230	0.349	1.93	1.65	5.44	3.77	89	0.16
	306	0.395	1.83	1.56	5.37	3.64	94	0.13

**Table 5:** Parameters extracted from SANS model fits (core-shell ellipsoid form factor + RMSA structure factor) of mixed SDS/BrijL23 surfactants in $D_{2}O$ at 20.0 ${}^{\circ }C$. For some solutions, fits with the RMSA structure factor do not converge and fitting is carried out using the HS-PY structure factor instead with no fractional charge β extracted.

*X*	*c*	ϕ	$x_{c}$	$t_{s}$	$\rho_{s}$·10${}^{-6}$	$r_{\mathrm{eff}}$	$N_{\mathrm{agg}}$	β
	mM			nm	Å${}^{-2}$	nm		
0.05	66.0	0.238	2.06	2.25	5.50	4.44	61	–
	82.5	0.280	1.98	2.20	5.47	4.36	61	–
	124	0.372	1.87	2.09	5.40	4.19	62	–
	168	0.437	1.73	2.01	5.37	4.05	64	–
0.11	66.3	0.223	1.99	2.27	5.51	4.43	65	0.08
	82.9	0.272	1.99	2.24	5.50	4.40	65	0.07
	124	0.381	1.96	2.11	5.46	4.25	63	–
	165	0.436	1.82	2.01	5.38	4.09	66	–
0.21	66.9	0.219	1.90	2.28	5.58	4.40	65	0.15
	83.6	0.275	1.97	2.24	5.56	4.39	65	0.13
	125	0.372	2.02	2.15	5.56	4.32	69	–
	167	0.429	1.90	2.02	5.47	4.13	69	–
0.32	67.5	0.222	1.79	2.26	5.64	4.32	62	0.19
	84.4	0.268	1.85	2.22	5.66	4.32	64	0.17
	127	0.360	1.93	2.09	5.61	4.23	67	0.11
	169	0.416	1.89	2.00	5.57	4.11	71	–

**Table 6:** Parameters extracted from SANS model fits (core-shell ellipsoid form factor + RMSA structure factor; pure Tween20: HS-PY structure factor) of mixed SDS/Tween20 surfactants in $D_{2}O$ at 20.0 ${}^{\circ }C$ (* fixed values).

*X*	*c*	ϕ	$x_{c}$	$t_{s}$	$\rho_{s}$·10${}^{-6}$	$r_{\mathrm{eff}}$	$N_{\mathrm{agg}}$	β
	mM			nm	Å${}^{-2}$	nm		
0.00	142	0.240	2.14	1.78	5.08	3.99	95	–
0.06	143	0.257	2.17	1.80	5.14	4.03	92	0.06
0.12	146	0.270	2.17	1.82	5.21	4.05	90	0.10
0.24	149	0.278	2.12	1.78	5.33	3.99	86	0.16
0.35	153	0.268	1.96	1.71	5.47	3.85	82	0.20
0.43	174	0.231	3.15	1.83	5.67	4.43	166	0.12
0.54	177	0.215	2.89	1.67	5.77	4.17	150	0.13
0.64	179	0.150	1.73	1.08	5.33	3.12	91	0.24
0.73	182	0.104	1.77	0.80	5.15	2.84	100	0.24
0.82	184	0.111	1.57	0.78	5.33	2.74	86	0.28
0.91	187	0.096	1.50	0.65	5.29	2.58	84	0.32
1.00	180	0.090	1.72	0.69 *	6.34 *	2.71	99	0.27

**Table 7:** Parameters extracted from SANS model fits (core-shell ellipsoid form factor + RMSA or PY structure factor) of mixed SDS/BrijL23 surfactants in $D_{2}O$ at 20.0 ${}^{\circ }C$ with a total surfactant concentration *c* = 127 mM and a mixing ratio *X* = 0.32.

*S*(*q*)	ϕ	$x_{c}$	$t_{s}$	$\rho_{s}$·10${}^{-6}$	$r_{\mathrm{eff}}$	$N_{\mathrm{agg}}$	β
			nm	Å${}^{-2}$	nm		
RMSA	0.360	1.93	2.09	5.61	4.23	67	0.11
HS-PY	0.371	2.01	2.14	5.67	4.30	68	–

## Data Availability

Small-angle neutron scattering data are available from doi:10.5291/ILL-DATA.EASY-440 and doi:10.5291/ILL-DATA.EASY-646.

## Appendix A. Mass Spectrometry of Nonionic Surfactants

MALDI-TOF experiments were carried out using an Autoflex speed TOF/TOF (Bruker Daltonik) mass spectrometer. A saturated solution of alpha-cyano-4-hydroxy-cinnamic acid (HCCA) in acetonitril/0.1% aqueous TFA (30:70, *v*:*v*) was used as the matrix. The possible added salt solutions were 0.1 mol L${}^{-1}$ aqueous KTFA or NaTFA solutions. Aqueous solution of Tween20 and BrijL23 with a concentration of *c* = 100 μg mL${}^{-1}$ were prepared. A total of 20 μL of the aqueous surfactant solution was mixed with 20 μL matrix solution and 1 μL salt solution. Then, 1 μL of the resulting mixture was deposited on the sample target and dried. Measurements were performed operating in the positive-ion reflector mode. Spectra in the *m*/*z* range of 500 to 2500 were obtained by accumulating data from 4500 laser shots. The mass spectra were evaluated using the mMass software (Version 5.5.0). The instrument was provided by the German Research Foundation (DFG) through grant No. INST 163/445-1 FUGG (MALDI MS).

Figure A1 shows the measured mass spectra of Tween20 in different matrices. Peaks in the spectra can be described as a mixture of PME and IPE. The different peaks of each set are spaced by a mass/charge ratio of 44.03, corresponding to the mass of ethylene oxide (EO, $C_{2}H_{4}O$) unit.

Previous studies revealed that the commercially available Tween20 is often a mixture of various compounds [77,78,79,80,81,82]. The two major components found in this study are shown in Figure A2. In order to understand the occurrence of both compounds, the synthesis pathway of the surfactant has to be reviewed. Upon dehydration of sorbitol, which is typically the first step in the synthesis of polysorbate surfactants, a mixture of sorbitan and isosorbide can be obtained. Further steps in the synthesis (ethoxylation and esterification) then lead to the two compounds that were found in this study: polysorbate monoester (PME) and isosorbide polyethoxylate (IPE).

Table A1 summarises the key findings. In all measurements, PME as well as IPE components were identified. The aliphatic alkyl chain is predominantly $C_{11}$ with a small amount of $C_{13}$ chains also present. The number of EO units is roughly 25–26 for the PME molecules (w + x + y + z) and 10–11 for IPE molecules (p + m). Interestingly, the mean value over all PME and IPE molecules is approximately 20–22 which is similar to the value given by the manufacturer (=20). The Tween20 surfactants are, therefore, modelled assuming that the head-group consists of 20 EO groups.

**Table A1:** Key findings from mass spectrometry measurements of Tween20: the ratio between PME and IPE species, the ratio between $C_{13}$ and $C_{11}$ alkyl chains of the hydrophobic tail and the total number of EO groups per molecule: w + x + y + z of PME, p + m of IPE; ${}^{\left(a\right)}$ determined as the ratio of the peak areas from molecules with a $C_{11}$ alkyl chain; ${}^{\left(b\right)}$ determined as the ratio of the peak areas of PME.

Matrix	PME:IPE ${}^{\left(a\right)}$	$C_{11}$:$C_{13}$${}^{\left(b\right)}$	w + x + y + z	p + m
HCCA	4.5:1	3.3:1	25.7 ± 0.1	10.8 ± 0.1
HCCA + $K^{+}$	2.0:1	4.7:1	25.6 ± 0.1	10.7 ± 0.1
HCCA + ${\mathrm{Na}}^{+}$	5.7:1	6.1:1	25.6 ± 0.1	10.7 ± 0.1

Figure A3 shows the measured mass spectra of BrijL23 in HCCA matrix, with and without the addition of different salts. Similar to the case of Tween20, the peaks of different sets are spaced by the mass value of one EO group.

Table A2 summarises the two key findings. The alkyl chain length is predominantly $C_{12}$, with a small portion of $C_{14}$ chains present. The number distribution of EO groups between single surfactants can be described as Gaussian shaped. The mean value of EO group per surfactant is roughly 23, the value given by the manufacturer. This result confirms the findings from previous literature studies [77,80].

**Table A2:** Key findings from mass spectrometry measurements of BrijL23: the ratio between $C_{12}$ and $C_{14}$ alkyl chains of the hydrophobic tail and the total number of EO groups per molecule.

Matrix	$C_{12}$:$C_{14}$	EO Groups
HCCA	8:1	21.6 ± 0.1
HCCA + $K^{+}$	42:1	23.6 ± 0.1
HCCA + ${\mathrm{Na}}^{+}$	14:1	24.2 ± 0.1

## Appendix B. Surfactant Properties

Nonionic surfactants (BrijL23 and Tween20) are determined by density measurements (DM40, Mettler Toledo, Germany) of aqueous surfactant solutions at five different, known mass fractions. Densities of the nonionic surfactant molecules are then determined by linear regression. The molecular volume is then calculated, knowing the molar mass and the density. The following tables summarise the properties used to fit the small-angle neutron scattering profiles from dispersions of surfactant micelles.

**Table A3:** Critical micelle concentrations cmc, molar mass *M* as given by the manufacturer, the determined densities and the molecular volume $v_{m}$ of the used surfactants.

Chemical/Formula	cmc	*M*	Density	$v_{m}$
	mM	g mol${}^{-1}$	g cm${}^{-3}$	nm${}^{3}$
SDS/$C_{12}H_{25}SO_{4}\mathrm{Na}$	8.3 [83]	288.4	1.01	0.410
Tween20/$C_{58}H_{11}O_{26}$	0.059 [41]	1128	1.150	1.805
BrijL23/$C_{58}H_{118}O_{24}$	0.09 [9]	1198	1.115	1.809

**Table A4:** Molecular volumes $v_{m}$ are given in [57,84]. Neutron scattering length densities ρ of the surfactants alkyl chains $C_{11}H_{23}$ and $C_{12}H_{25}$, ethylene oxide (EO, $CH_{2}CH_{2}O$) contained in the headgroups, and the solvent $D_{2}O$ are calculated with the NIST NCNR SLD calculator, knowing the molecular volume $v_{m}$ and the molar mass *M* (www.ncnr.nist.gov, accessed on 5 May 2021).

Group	$v_{m}$	ρ
	nm${}^{3}$	10 ${}^{-6}$ Å ${}^{-2}$
$C_{12}H_{25}$	0.351	−0.39
$C_{11}H_{23}$	0.324	−0.39
$CH_{2}CH_{2}O$	0.0616	0.67
$D_{2}O$	0.0301	6.34
